# "I will not let my HIV status stand in the way." Decisions on motherhood among women on ART in a slum in Kenya- a qualitative study

**DOI:** 10.1186/1472-6874-10-13

**Published:** 2010-04-28

**Authors:** Opondo Awiti Ujiji, Anna Mia Ekström, Festus Ilako, Dorcas Indalo, Birgitta Rubenson

**Affiliations:** 1 Karolinska Institute, Department of Public Health Sciences, Division of Global Health, SE- 171 77 Stockholm, Sweden; 2 African Medical Research Foundation in Kenya, P.O. Box 30125-00100, Nairobi, Kenya; 3 AMREF Kibera clinic, P.O. Box 30125-00100, Nairobi, Kenya

## Abstract

**Background:**

The African Medical Research Foundation antiretroviral therapy program at the community health centre in Kibera counsels women to wait with pregnancy until they reach the acceptable level of 350 cells/ml CD4 count and to discuss their pregnancy intentions with their health care providers. A 2007 internal assessment showed that women were becoming pregnant before attaining the 350 cells/ml CD4 count and without consulting health care providers. This qualitative study explored experiences of intentionally becoming pregnant among women receiving highly active antiretroviral therapy (HAART).

**Methods:**

Nine pregnant women, six newly delivered mothers and five women wanting to get pregnant were purposefully selected for in-depth interviews. Content analysis was used to organize and interpret the women's experiences of becoming pregnant.

**Results:**

Women's choices for pregnancy could be categorized into one overarching theme 'strive for motherhood' consisting of three sub-themes. A child is thought of as a prerequisite for a fulfilled and happy life. The women accepted that good health was required to bear a pregnancy and thought that feeling well, taking their antiretroviral treatment and eating nutritious food was enough. Consulting health care providers was perceived as interfering with the women's decisions to get pregnant. Becoming pregnant as an HIV-infected woman was, however, complicated by the dilemmas related to disclosing HIV infection and discussing pregnancy intentions with their partners.

**Conclusions:**

Motherhood is important to women on antiretroviral treatment. But they seemed to lack understanding of the relationship between a high CD4 cell count and a low chance of transmission of HIV to offspring. Better education about the relationship of perceived good physical health, low CD4 cell count and the risk of mother to child transmission is required. Women want to control the domain of childbearing but need enough information to make healthy choices without risking transmission.

## Background

An internal assessment in 2007 (unpublished results) at African Medical Research Foundation (AMREF) community health centre which provides free antiretroviral therapy (ART) in the Kibera slum in Nairobi, showed that 64 out of the 468 women on antiretroviral therapy conceived when their CD4 cell count was below 350 cells/ml, against all recommendations and without receiving any preconception counseling. Women enrolled on antiretroviral therapy at the AMREF community health center are told that they can have children with a high chance to be born free of HIV if the correct precautions are taken, and women are urged to discuss their pregnancy intentions with health care providers to enable pre-conception assessment and plan for prevention of mother-to-child transmission of HIV (PMTCT). Women on antiretroviral therapy are counseled and advised to wait with pregnancy until HIV is suppressed and a stable CD4 count of 350 cells/ml is reached. In addition the counseling stresses consistent condom use to reduce the risk of transmitting the virus and sexually transmitted infections (STIs) to their partners and having timed unprotected sex during the most fertile days. Studies have shown that persons living with HIV continue having a sexual life after diagnosis [1-3]. Many want to have children and have intentional pregnancies [4,5]. The optimum levels of CD4 cell count and timed conception significantly reduce the risk of transmitting HIV to both a partner and an unborn child [6,7]. It was thus important to understand issues regarding becoming pregnant among women on antiretroviral treatment.

The value put on children varies between societies and depend on the functions and needs they fulfill for both parents and community [8-10]. In African societies children ensure the continuity of the family lineage, confer a sense of continuity and inherit family land and wealth [11-14]. The highly regarded funeral traditions may also be affected by the fertility status of the deceased. Thus children safeguard a proper burial for their parents [13,14]. For the individual, an HIV diagnosis is thus not only associated with fear of death, but also with anxiety caused by the realization that one may die without being married or having children [15]. Childlessness and infertility are very stigmatizing and often associated with profound negative social repercussions in African societies [13]. Women are usually blamed for childlessness [16] and regardless of HIV sero-status, they therefore want to realize motherhood. With the scale-up of antiretroviral therapy in Kenya, women living with HIV have the opportunity to bear children with a greatly reduced risk of mother to child transmission.

However, very little is known about why women with a low CD4 count may choose to become pregnant despite a higher risk of MTCT and pregnancy-related morbidity, and without consulting health care providers. In this article, we explored the reasons behind this behaviour, aiming to inform and adapt service provision to better meet the needs of HIV-positive women and increase the uptake and coverage of PMTCT and safe motherhood monitoring. The aim of this study was to explore experiences of intentionally becoming pregnant among women receiving highly active antiretroviral therapy (HAART).

## Methods

### Study area

The Kibera Community Health Centre supported by AMREF, a non-government organization (NGO) is situated in the Kibera slum in Nairobi. It is estimated that about 60 percent of Nairobi residents live in slums [17]. The Kibera slum, which has between 0.5-1 million people, attracts migrants from rural areas in search of employment and livelihood [18]. The existence of many entertainment spots providing alcohol, drugs and large networks of commercial sex workers make Kibera a high-risk environment for HIV transmission [19]. The exact figure for HIV prevalence in Kibera is unknown, but it is estimated to be higher than the national prevalence of 7% [20]. Kibera has an inadequate coverage of health care services and inhabitants depend on informal health care providers and a few primary health care facilities run by NGOs. Men engage in a wider range of informal jobs than women who often perform domestic work or petty trade. Kibera constitutes a highly mobile and sexually active young population living closely together in deplorable conditions and interacting in loosely structured sexual relationships [21]. Although men and women may live in permanent unions, it is hard to predict sexual exclusivity and monogamy in these relationships [22].

### The antiretroviral therapy program at the Kibera Community Health Centre

The Kibera health centre offers preventive, diagnostic and basic health care, including services focusing on immunization and reproductive health. An antiretroviral therapy program was started in 2003 and provides free treatment and care, including home based care and adherence support for HIV-infected individuals. The clinic also provides free voluntary counseling and testing (VCT) and HIV prevention of mother to child transmission (PMTCT) services. Initially, antiretroviral therapy enrolment was based on the world health organization (WHO) clinical staging only, but CD4 and viral load testing were later introduced. In 2006 the routine use of viral load was discontinued due to economic restraints but when needed the samples are sent to the national reference laboratory, at the Kenya National Medical Research Institute (KEMRI), located at the national referral hospital Kenyatta. The first line treatment consists of Nevirapine, Lamivudine, Stavudine or Zidovudine. As second line treatment Abacavir, Didanosine, Lopinavir or Tenofovir is used. No third line treatment is available.

Counselors and post-test clubs (PTCs) support adherence and retention in the antiretroviral therapy program. Additional support is provided through treatment literacy training for children and adults, social assessments and change of pill-regimes to fixed doses. At the time of antiretroviral therapy enrolment, social workers record personal and contact information of patients to enable for tracing in the event that they default. Tracing of defaulting patients is done by community health workers with support from PTCs. Patients are followed up every three months by a clinical officer to assess adverse drug effects, clinical staging and adherence to antiretroviral therapy, and to replenish their antiretroviral medicines. CD4 count is performed every six months with the exception of pregnant women who undergo routine testing every three months. As of January 2009 the antiretroviral therapy program had enrolled 2535 adults of whom 1326 were on antiretroviral treatment. The health centre had six clinical officers, 19 nurses, four midwives and two staff each for counseling, nutrition-advice and VCT.

### Study design

This study employed qualitative methods aimed at uncovering, explaining and giving meaning to human behaviour from the perspective of those being studied. Qualitative interview methods facilitate rapport while enabling the interviewer to maintain focus on the issue at hand [23,24].

The research team consisted of a Kenyan sociologist (OAU) residing in Sweden, a Kenyan physician and epidemiologist (FI), a Swedish medical doctor and epidemiologist specialized in HIV and AIDS (AME), a Swedish public health scientist (BR) working in the field of HIV and AIDS and a Kenyan social worker (DI). No one in the team worked clinically with the patients at the antiretroviral therapy clinic. While in Kenya, OAU worked with projects on HIV/AIDS prevention and control focusing on gender and sexuality interventions. Her background in combination with her experience from field work and her public health perspective bring knowledge and contextual understanding to the study.

### Sampling of informants

The informants were purposively selected from the registers at the antiretroviral therapy and PMTCT programs. The criterion was that the women had been on HAART for not more than six months and the theoretical assumption was that their experiences of becoming pregnant would differ depending on the time-line of pregnancy. Through the registers, it was possible to identify three different groups. The first two groups were located in the registers then retrospectively identified and the third group was prospectively identified. The groups were: (i) pregnant women (ii) women who had delivered in the past 12 weeks and (iii) women who were seeking to become pregnant. Ten were pregnant, eight had delivered and five were seeking a pregnancy. A community health worker sought the help of a female leader of the post-test club to identify women attempting to become pregnant and enrolled in antiretroviral therapy at the Kibera community health centre.

One community health worker contacted all the groups of women and introduced them to the study. The women willing to participate provided verbal consent and were asked to indicate the time and place of the interview. The interviews were performed at the clinic or home within three days of providing informed consent. No one declined to be interviewed. However, three women dropped out for other reasons.

### Data collection

The main author performed all the interviews. A semi-structured question guide (additional file 1) was used during the interviews, which were tape recorded with the participants' permission, and conducted in Kiswahili. We asked the women to describe freely and reflect on pregnancy in general and how they prepared for and achieved it. We used probing during the interviews to explore motivations for pregnancy, the women's role in achieving it, pregnancy discussion with the partner, HIV status disclosure, and interaction with medical providers. Positive and negative experiences and perceived problems and challenges in seeking childbearing during antiretroviral therapy were also probed. Initially one woman from each of the three groups was interviewed. A preliminary analysis was conducted by OAU and DI to provide insights and a chance to improve the flow and clarity of the questions. The women were not given any incentives or rewards. Scenarios that could arise during interviews were discussed with a nurse, social worker and counselor in advance to prepare for situations of emotional distress that could arise. The staff shared their experiences and gave OAU ideas on how to handle situations that required emotional assistance. A counselor was available for referral for women who needed support.

### Data analysis

**Figure 1:**
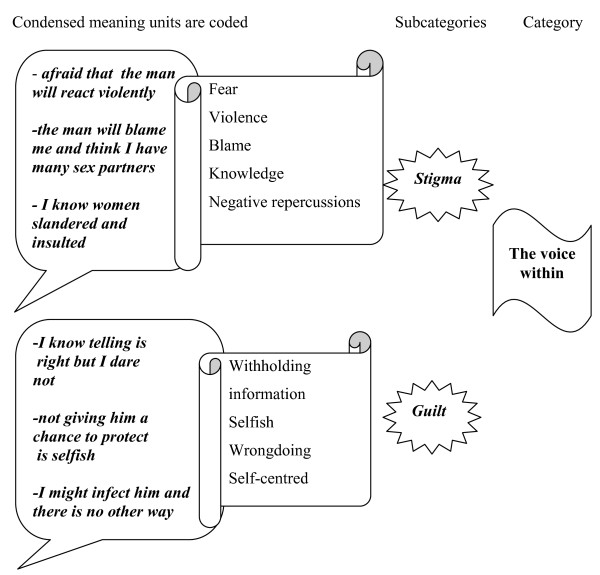
**Coding process from meaning units to category**.

Data were analyzed qualitatively using content analysis, guided by Graneheim and Lundman (2004). Initially, the transcribed material was read a number of times to get a general sense of the material. Meaning units, which are key phrases in the text, were identified, condensed and outlined. Codes were then ascribed to each meaning unit. The codes were then compared and grouped into sub-categories. This comparison was performed consistently to identify emerging categories that were further compared, re-organized and merged into sub-themes and one overarching theme. The coding and analysis were first carried out by the OAU independently and then together with AME and BR. Sub-categories, categories and themes were arrived at by consensus between the authors to describe the women's experiences of becoming pregnant of the women. An example of the coding procedure is shown in figure 1.

### Trustworthiness of the study

Trustworthiness is related to the ability of the study method to capture the reality of those being studied. In this study, OAU's prolonged engagement in understanding the functioning of the antiretroviral therapy and PMTCT programs at the AMREF Kibera health centre, her participation in field visits and data collection activities were crucial. Using face-to-face interviewing enabled the interviewer and informant to build rapport and discuss issues freely. Peer debriefing sessions and joint analysis by the research team, who represented different disciplines and experiences, were important to increase the credibility of the study results.

### Ethical Considerations

Ethical approval was obtained from the Kenya Medical Research Institute (KEMRI) and Karolinska Institute. KEMRI has an ethical review committee, which vets all research proposals that involve humans. The committee is accepted by the Ministry of Health as a National Ethical Review Committee. All study participants were assured that their anonymity would be strictly upheld throughout and after the study period. It was stressed that participation in the study was voluntary, and participants could withdraw at any time with no effect on them, their family or care and treatment given. Each woman gave a verbal informed consent because of their high illiteracy levels and reluctance to have a written record of their signatures on the informed consent form. Interviewing well-informed women living with HIV about their sexual behaviour, including unprotected sex and non disclosure of HIV infection, requires great sensitivity not to impose on the women's integrity or confuse the role of researcher with that of a counselor [23].

## Results

### Characteristics of the study population

In total 50 women were eligible to participate in the study. Twenty-three had been invited to participate and 20 were available for interviewing. The informants were aged between 22 and 45 years. Sixteen had primary school education and all were Christians. Nine of the women were pregnant; six had delivered within the last eight weeks and five were seeking a pregnancy. Six women were single, six cohabiting, four married and four widowed. All women had disclosed to their mother, sister or aunt. Six women had disclosed to their partners. The women's parity ranged from two to five. None of participants had a regular income.

### A theme on becoming pregnant

**Figure 2:**
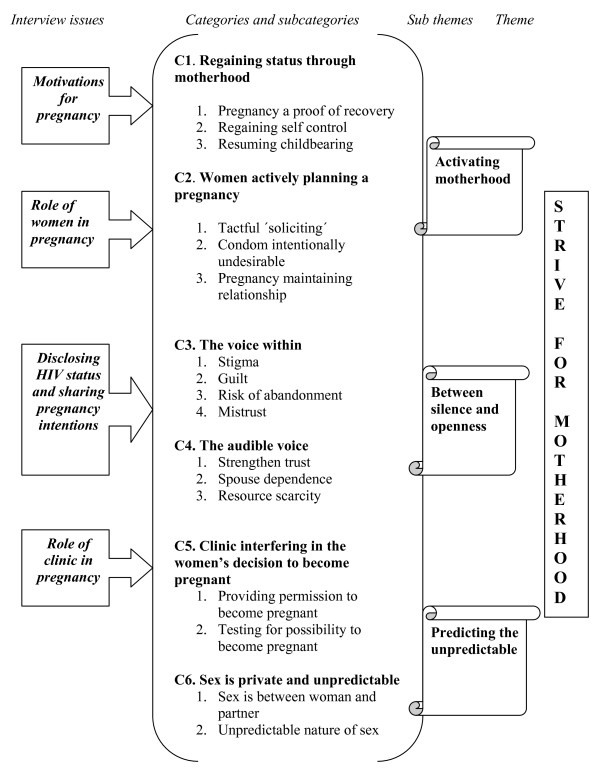
**'Strive for motherhood' - a theme on becoming pregnant when CD4 is low**.

One theme 'strive for motherhood' consisting of three sub-themes emerged from the women's descriptions about reasons for becoming pregnant despite a low CD4 cell count. Each sub-theme illustrates a different aspect in the process of achieving a pregnancy (Figure 2). 'Activating motherhood' refers to women's explanation for pregnancy and their role in achieving a pregnancy, while the theme 'Between silence and openness' points to the dilemmas that women face in their decision to share or not to share their HIV infection and pregnancy intentions with their partners. 'Predicting the unpredictable' is an illustration of how the role of the clinic in pregnancy is not meeting the needs of the women when they wanted to become pregnant. The emergence of the theme 'strive for motherhood' is shown in figure 2.

### Activating motherhood

The women wished to regain self-status and that was achievable through motherhood. Pregnancy was pursued as a proof of recovery and evidence of regained self-control.

*Before I was falling sick........ I am recovered now. What is important....... is to eat properly to have energy and be strong. To carry it [pregnancy] needs strength and it [childbirth] tests........the body. I became pregnant........ when I could cook and wash for myself........there was no one to help me here [Kibera]. I had to be able to take care of myself ........just as I did before my illness*. (Pregnant and widow 30 years old)

The women knew that children would also improve their status in their original family and make them recognized. They described a child as a proof of the presence of a man in their lives. This link was evidence of them being attractive and capable of seducing a man. Women without children often lacked acceptance.

The women underscored the importance of establishing a relationship with a man when seeking a pregnancy. They employed tactful 'soliciting' to attract men and played an active role in pursuing and keeping a man. The women knew that men perceived them as sex providers and they used sex to lure men into a relationship.

*All they [men] see in us [woman] is sex...... I just dress smart and have a nice hairstyle for them [men] to see me.......he [man] will surely look and want me. .......I will flirt and show interest*. (Single 38 year old planning a pregnancy)

The women described how condoms were intentionally undesirable when they wanted to conceive. They explained that men did not want to use condoms after a few encounters and that improved their chances of becoming pregnant. Seeking a pregnancy after previous sex encounters was preferred as condom use subsided.

Children were vital in uplifting the women's image and were always welcomed by the women. Women in stable relationships reported that they would also get a child to make the man happy and maintain the union, and also opted for a pregnancy to meet a partner's need to name a child after family members or to consummate a new relationship.

*My husband treats me well........gives me money........ to buy food and clothes.....he [husband] pays the rent..... I will get pregnant......he wants to name after his father, mother or any person he loves....he will be happy...He will always be concerned for it [child]......the child has replaced his relative.......it [child] is a living reminder.........I will take care of him and the child*. (Delivered and married 34 year old)

*I have been seeing him [partner] for two years [in the past]....he asked me to bear him a child......I happily agreed......I already have two [children]...... from before [previous relationship].......it showed me that he [partner] loves me........he [partner] wanted me to have a child........his own blood to bond us ....he was serious about staying with me...... and build a family*. (Pregnant and cohabiting 28 year old)

Delaying to achieve a pregnancy could disrupt a much needed relationship.

*It was not my intention for it [pregnancy].....my husband wanted another child.....it took four months [to conceive]....my husband kept asking.....if I was using them [birth control pills] but I wasn't .....he was very impatient......he threatened me....... he will chase me away...... if I could not get it [pregnant]*. (Pregnant and married 34 year old)

### Between silence and openness

Two dilemmas were encountered in their quest for becoming pregnant: (i) fear of disclosing their HIV status, and (ii) not being able to discuss their desire for a pregnancy openly with their partners. The women expressed guilt of possibly infecting the partner, but also feared that revealing their HIV status could expose them to stigma and the risk of abandonment by the possible partner: thereby minimizing their chance of getting pregnant. Even though the women acknowledged that disclosure to a sexual partner was vital, many concluded they would still keep this information secret to maintain the possibility of being in a relationship and becoming pregnant.

*I know telling him [partner] about it [HIV infection] is right......I choose not to......I look bad for thinking of me .... he [partner] can get that [HIV] from me but you need to understand my situation [want a child]......I cannot get it [pregnant] if he leaves or chooses to use a condom*. (Widow 33 years old and planning a pregnancy)

Non-disclosure also ensured that the woman could be in a relationship with another man if the current relationship did not last. Not being identified as HIV infected made it possible for them to approach men and men to be around them. This created an opportunity for women to find another man.

*I will never get a man here [in Kibera].....if I am known to have HIV. No man will want me [to befriend]....not even seen together as friends. He [man] will be teased and laughed at........I will be an embarrassment.....and no pride to a man*. (Pregnant and single 26 year old)

The women feared that a discussion about pregnancy intention could be seen as trying to impose on the man.

*I do not reveal that [pregnancy intentions]....he [man] will think that I want to give him responsibility [a child]...... or trap him to marry me.....he [man] may get frightened [of pregnancy]......and insist on using a condom...... or just leave me*. (Delivered and cohabiting 25 year old)

When asked how they perceived disclosing and discussing pregnancy, women often described a time process. They initially wanted a child and feared not having a man to make them pregnant. After getting the child, the women were prepared to disclose and discuss a pregnancy when they wanted marriage.

*The most important thing..... I want to prove my capability as a woman [childbearing]...... I want to be recognized in my family and community...I will not let my status stand in the way......... I will not share it with him [partner] if he [partner] will not support me.......I can worry about the other things [love and marriage] afterwards*. (Delivered and married 44 year old)

All women felt that disclosure could strengthen partner trust as it showed their openness, encouraged partner support and improved communication. However, this happened only after the women had satisfied their desire for a child and did not fear losing the man. They felt that discussing pregnancy intentions for additional children was vital because resources were scarce and there was a need to plan for the future of an expanding family.

### Predicting the unpredictable

When asked how they felt about becoming pregnant without involving the clinic, the women expressed indifference. Pregnant women and the newly delivered mothers did not regret their action. They felt that the clinic had no role in planning a pregnancy. All women expressed the willingness to have a healthy pregnancy, but they perceived the role of the clinic as interfering in their decision to become pregnant. They felt that the recommendation to wait with pregnancy until the required level of CD4 cell count was reached could delay the pregnancy. They felt that the clinic was restricting them and expecting them to ask for permission to become pregnant.

*They [clinic] want me to wait [pregnancy] yet I am ready [healthy]. I see no need for them [clinic] to know when I want it [a child]......it [child] is mine and I get one when I want*. (Cohabiting 33 year old planning a pregnancy)

The women felt that the clinic was taking tests to make a decision on their capability to become pregnant and carry a pregnancy. For some of the women, the testing was perceived unnecessary.

*They [clinic] want to check me [woman's health] before becoming pregnant.....I will not go for that [CD4 count]. I know and experience it [good health].....I live in this body. They [clinic] cannot tell me what I feel....it is the other way round [woman tells the clinic how she feels].....tests want to tell me what I already know*. (Pregnant and cohabiting 37 year old)

The women acknowledged being treated well and welcomed by the counselors. They expressed satisfaction with their providing information about childbearing when living with HIV. The women hinted that this information was obtained also from PMTCT advertisements. Women who had given birth to children free of HIV provided testimonies in TV-shows or on billboards that antiretroviral therapy is effective and bearing healthy children is a possibility. This information gave the women hope and inspired them to seek a pregnancy, but it did not mention discussing pregnancy intentions with the clinic or that a pregnancy needed special tests. That treatment was free and accessibility was described as important. In addition the advertisements underlined that PMTCT programs existed just to serve HIV-infected women like them who were pregnant.

*I can get it [baby]......the treatment [antiretroviral therapy] has made me feel better.....look at Lucy (pseudonym) [famous Kenyan HIV and AIDS].....she was on the television.......talking about her pregnancy...... she [Lucy] got a healthy child after taking medicine [antiretroviral therapy]*. (Delivered and married 34 year old)

When asked how they felt about the advice to have timed unprotected sex, the women expressed resentment. They described sex as a private and unpredictable act. Others felt that unprotected sex did not just happen, but it was a desire difficult to dictate.

## Discussion

This study highlights the various decisions faced by the women on ART in the urban slum of Kibera, from deciding for a pregnancy through to achieving it, and the determination to actively plan to control their lives in the process.

The findings show that in this urban slum setting, women became pregnant by 'activating motherhood', i.e. actively planning and strategizing to get pregnant and having children. A child gave them identity and recognition in the community, and thus happiness and fulfillment as women. Their motivation for a pregnancy seemed to depend on the perceived meaning of a child and the importance of bearing one's own child. These findings are consistent with other studies performed in African societies which show that the identity of a woman is pegged on her capacity to bear children and that children are equated to a fulfilled life as a woman [14,25]. As a result of the increased antiretroviral therapy availability women saw a possibility to restore their status through motherhood also shown in other studies [26,27]. Our study has gone a step further to also reveal the meaning of pregnancy as a sign of recovery from ill health. The capability to perform a woman's duties including resumption of child bearing was a means of obscuring the HIV-infection and assuming the same status as women without the virus.

Regained health was interpreted as feeling strong and not falling sick - a sign that HAART is working [27,28]. For the women being strong enough to take care of themselves and performing normal duties were signs of the good health permitting for seeking a pregnancy. They did not understand that physical improvements were often experienced before the recommended laboratory health markers were achieved. This perception gap between laboratory health markers and individual health indicators should be addressed during counseling, so that women understand how it relates to their childbearing goals. The women required better education on the relationship between low CD4 count and high risk of HIV transmission from mother to child. The informants in this study, just as many residents of Kibera have low education levels. Counseling could therefore be clearer and simplified to meet the needs of illiterate and semi-illiterate women. Women want to control the domain of childbearing but need enough information to frame the choices in a way that still makes them feel empowered. They want to bear healthy children but felt the preconception care was attempting to 'predict the unpredictable'. To them becoming pregnant was a private desire that could eventually include their partners but where clinic staff were outsiders. The preconception counseling was poorly matched to the contextual structures influencing how women achieved a pregnancy, i.e. how women and men established sexual relationships. As the PMTCT advertisements did not explicitly mention counseling or the need to monitor CD4 cell counts to optimize the timing for pregnancy, such components were met with resentment and skepticism.

The women were tactical when establishing a relationship that made it possible to have sex without a condom. Their role in planning a pregnancy was based on their knowledge of men's behaviour towards sex. The reluctance to use a condom among men was exploited by the women who knew that after a few encounters the condom would be disregarded and disappear. Other studies have shown how the use of male condoms varies with partner types and that it is lowest with regular or married partners [29,30]. Although the women regarded conceiving within established relationships as preferable, they faced the dilemma 'between silence and openness', disclosing their HIV status or not, discussing pregnancy intentions or not. Family members particularly females seemed to be more preferred and trusted when disclosing a positive HIV status, which has also been shown in another in a study from Nairobi [31]. The fear that their partners could react negatively to the HIV diagnosis and leave them without a man to conceive with was a big concern. Similarly a study in Tanzania found that reactions of male partners to an HIV-diagnosis was a major concern for women [32].

Discussions with the partner about a possible pregnancy were also avoided as the women feared it might encourage condom use or that the man would leave. However women also told about men who were eager to have children and how they accepted becoming pregnant to maintain a relationship that seemed permanent. The possibility that the partner might want a child made women in stable relationships always anticipate childbearing. The decision to disclose HIV infection means weighing enormous potential consequences, both positive and negative. The women in this study expressed guilt for not giving their partners a chance to decide upon condoms. However, the need to get pregnant and attain status was more important to them than to protect a partner and remain unfulfilled and unhappy. When the desire for childbearing was at stake, the rewards of transparency were outweighed by the risk incurred. The women might choose to disclose when their need for children is satisfied.

### Methodological Considerations

The fact that OAU had been seen at the health centre for a period of time before the interviews may have influenced her relations with the respondents. It was thus important to underline her role as a researcher and that what they said would not affect their access to health care at the facility. OAU's pre-knowledge about the context and the topic were mainly an asset to the study, but it was also important to at times put it within brackets to allow for unanticipated findings.

Our study explored how women intentionally become pregnant and their reasoning around it. Issues on contraceptive use were mentioned in the interviews and will be discussed elsewhere, as they had no prominent role when pregnancy was desired. The study could have benefitted from understanding the relationship of the women with their partners when a pregnancy was not the goal, but as this was not discussed during the interviews it is not part of the study. Also the importance a pregnancy and a child ascribe to the status and acceptance of women in the Kenyan society made it so obviously part of any relationship and that is where the interviews focused. None of the women mentioned being engaged in transactional sex for survival, which may be a result of the selection of the women through the antiretroviral therapy program and the focus on pregnancy and childbearing. Further studies are needed that explore the interplay between men's relationship patterns and pregnancy needs particularly how cohabiting women manage to hide their status when on HAART.

Even if the findings in this study refer to the women participating, the desire for motherhood and the loose societal control making a pregnancy possible are factors true for many women in urbanizing Africa.

## Conclusion

Motherhood is important to women on antiretroviral treatment. But they seemed to lack understanding of the relationship between a high CD4 cell count and a low chance of transmission of HIV to offspring. Better education about the relationship of perceived good physical health, low CD4 cell count and the risk of mother to child transmission is required. Women want to control the domain of childbearing but need enough information to make healthy choices without risking transmission.

## Competing interests

The authors declare that they have no competing interests.

## Authors' contributions

OAU is the main author of the manuscript and involved in all aspects of the study. BR and AME provided scientific expertise and feedback throughout the development of the study and manuscript. FI was involved in the conceptualization of the idea, tool development and reading and editing of the manuscripts. DI was involved during preparations and pre-test of the interview guide, data collection and reading and editing of the manuscript. All co-authors have seen and approved the final version of the paper and have agreed to its submission for publication.

## Pre-publication history

The pre-publication history for this paper can be accessed here:

http://www.biomedcentral.com/1472-6874/10/13/prepub

## Supplementary Material

Additional file 1 **Interview guide**. Questions asked to study participants to obtain information on intentionally becoming pregnant. Click here for file
