# Supplementary material for: "I will not let my HIV status stand in the way." Decisions on motherhood among women on ART in a slum in Kenya- a qualitative study
Source: BMC Womens Health. 2010 Apr 28;10:13. doi: 10.1186/1472-6874-10-13 (PMC2873237; doi:10.1186/1472-6874-10-13)
Supplement: Additional file 1 — Interview guide. Questions asked to study participants to obtain information on intentionally becoming pregnant. [file 1472-6874-10-13-S1.DOC]

**Pregnancy during ART**

1. How does pregnancy fit in your life?

Probe:

What is the importance of pregnancy?

What is the meaning of becoming pregnant?

How has a HIV diagnosis changed attitudes towards pregnancy?

1. How do you reason around becoming pregnant?

Probe:

What do you think about?

How do you prepare?

What do you do in becoming pregnant?

How does a HIV diagnosis fit into becoming pregnant?

1. How do you become pregnant?

Probe:

Who do you discuss a possible pregnancy with?

Who do you disclose to?

Who is involved in the seeking of pregnancy?

1. How does the clinic fit in you becoming pregnant?

Probe:

What are you told about pregnancy when on ART?

What do you make of preconception assessment care?

What do you feel about the safe timing of unprotected sex for pregnancy?

1. How do you see childbearing when living with HIV?

Probe:

What are the positive and advantageous issues?

What are the negative issues and challenges?

What do you suggest as ways for improving pregnancy occurrence among women on ART?
